# Ranges, temporal trend, and determinants of novel obesity indices in middle-aged and older Chinese population: insights from a nationally representative and longitudinal study

**DOI:** 10.3389/fpubh.2025.1571254

**Published:** 2025-08-14

**Authors:** Liufang Huang, Yifan Zhou, Kaiweisa Abuduxukuer, Chuchu Wang, Ya’nan Hou, Wenming Shi, Jianfeng Luo, Jin Wei, Yuhang Yue

**Affiliations:** ^1^Division of Pediatric Gastroenterology and Nutrition, Xinhua Hospital Affiliated to Shanghai Jiao Tong University School of Medicine, Shanghai, China; ^2^Department of Ophthalmology, Shanghai Tenth People’s Hospital, School of Medicine, Tongji University, Shanghai, China; ^3^Shanghai Municipal Center for Health Promotion, Shanghai, China; ^4^Department of Biostatistics, School of Public Health, Fudan University, Shanghai, China; ^5^NHC Key Laboratory of Health Technology Assessment, Fudan University, Shanghai, China; ^6^Key Laboratory of Public Health Safety of Ministry of Education, Fudan University, Shanghai, China; ^7^Department of Endocrine and Metabolic Diseases, Shanghai First People’s Hospital (Shanghai General Hospital), School of Medicine, Shanghai Jiao Tong University, Shanghai, China; ^8^School of Public Health, Fudan University, Shanghai, China; ^9^Department of Ophthalmology, Shanghai Municipal Hospital of Traditional Chinese Medicine, Shanghai University of Traditional Chinese Medicine, Shanghai, China; ^10^Department of Pediatric Neurology, Xinhua Hospital Affiliated to Shanghai Jiao Tong University School of Medicine, Shanghai, China

**Keywords:** CHARLS, obesity index, weight-adjusted-waist index, lipid accumulation product, visceral adiposity index, waist-to-height ratio, Chinese visceral adiposity index

## Abstract

**Background:**

Traditional obesity assessment using body mass index (BMI) fails to adequately capture fat distribution, particularly central obesity, which is closely linked to metabolic dysfunction and obesity-related complications. Alternative obesity indices that better reflecting fat distribution and body composition have shown promise, yet large-scale population-based data remain limited. This study evaluates the ranges, temporal trends, and associated factors of novel obesity indexes in a nationally representative cohort of middle-aged and older Chinese adults.

**Methods:**

Using data from the China Health and Retirement Longitudinal Study (CHARLS), we analyzed 17,708 adults in 2011 and followed 5,078 participants over 5 years nationally. Six obesity indexes: waist-to-height ratio (WHtR), lipid accumulation product (LAP), visceral adiposity index (VAI), body shape index (ABSI), Chinese visceral adiposity index (CVAI), and weight-adjusted waist index (WWI), were evaluated for ranges, temporal trends, and associated factors using logistic regression and generalized estimating equations (GEE). Associated factors included demographic, socioeconomic, lifestyle, and medical variables.

**Results:**

The national ranges were as follows: VAI (−1.805 to 6.615), ABSI (0.071 to 0.095), WHtR (0.462 to 0.622), CVAI (48.993 to 147.057), WWI (10.264 to 12.144), and LAP (−3.158 to 80.818). Significant associated factors were grouped into demographic (age, sex, urban/rural residence), medical (diabetes, and hypertension), and lifestyle factors (smoking, and drinking), with varying impacts across indexes. Over the five-year period, WHtR, CVAI, and LAP increased significantly (*β* = 0.00489, *β* = 4.51607, *β* = 6.37441; all *p* < 0.001), while ABSI decreased (*β* = −0.00048, *p* < 0.001). Interaction effects showed that diabetic participants experienced reductions in VAI, WHtR, CVAI, and LAP, indication a time-dependent change. In contrast, chronic kidney disease (CKD) and hypertension had stable effects on obesity indices, with no significant changes over time.

**Conclusion:**

This study provides nationally representative baseline ranges, temporal changes and influencing factors of novel obesity indexes among middle-aged and older Chinese adults. These findings underscore the potential of these obesity indices to guide clinical interventions aimed at controlling and preventing obesity-related health issues.

## Introduction

Obesity has become a major public concern due to its long-term health impacts and burden on healthcare systems, with its prevalence steadily rising worldwide (1). Traditionally, obesity has been assessed using BMI, a simple and widely used index for general adiposity (2). However, increasing evidence has revealed that higher BMI levels are paradoxically associated with better health outcomes or lower mortality risks in certain populations, particularly among those with chronic conditions. One major limitations of BMI is its inability to account for body composition (fat or lean mass), metabolic health status, and fat distribution such as abdominal and visceral adiposity (3, 4), which are now recognized as more clinically predictors of type 2 diabetes mellitus (T2DM), hypertension, and cardiovascular diseases (CVDs) than overall adiposity (5). For instance, in aging populations, body fat tends to redistribute toward the abdominal region, accompanied by progressive loss of muscle mass (sarcopenia) (6). In individuals with diabetes, a similar pattern may occur, whereby fat mass increases despite stable or declining BMI, primarily due to concurrent muscle loss (7). Moreover, BMI may underestimate body fat in taller individuals (8), and fail to distinguish between metabolically healthy obesity (MHO) and metabolically unhealthy obesity (MUO)—two phenotypes that differ substantially in their associations with cardiometabolic diseases such as T2DM and CVDs (9). This further underscores the heterogeneity in health outcomes among individuals with similar BMI values and emphasizes the need for more nuanced measures of adiposity and metabolic risk.

In recent years, alternative adiposity indices have emerged as more precise and non-invasive tools for assessing obesity-related health risks. These indices are derived from readily obtainable anthropometric and biochemical parameters and have demonstrated improved correlations with visceral adiposity and metabolic dysfunction compared to BMI (10). Waist-to-height ratio (WHtR), which relates abdominal fat to overall body height, offers a simple and effective global threshold (<0.5) for predicting metabolic and cardiovascular abnormalities without considering body weight (11). ABSI was created to compensate for the limitations of WC. It identifies individuals with the same weight but different waist sizes, predicting pathological risks that BMI cannot easily recognize, especially in individuals with normal BMI but central adiposity (12). Proposed in 2018, WWI has been proven more valuable in distinguishing between body fat and muscle, primarily addressing the issue of central obesity regardless of overall body weight. WWI is particularly suitable for older adults and individuals with sarcopenia, providing a more precise evaluation of central obesity risks (13). LAP, calculated as the product of WC and plasma TG levels, has been suggested as a reliable measure for evaluating the risk of insulin resistance (IR), MetS, and CVD (14). Similarly, the VAI—derived from WC, BMI, TG, cholesterol, and HDL—serves as an indirect indicator of visceral adiposity and provides insight into IR (15, 16). CVAI, specifically tailored for Chinese populations, modifies the VAI to account for ethnic differences in fat distribution, improving its predictive accuracy for MetS and T2DM (17). These indices show strong associations with abdominal fat and offer more reliable measures for predicting obesity-related disorders than BMI (18–22).

To date, the application of traditional BMI has been well-established in both research and clinical practice, with commonly accepted reference ranges (e.g., 18.5–23.9 kg/m2 for Chinese adults) based on national guidelines and large-scale epidemiological studies (23). With the growing recognition of novel adiposity indices in clinical research, there is an urgent need for large-scale, population-based studies to systematically investigate their reference ranges, distribution patterns, and associated determinates. China, as the most populous developing country, is facing a rising burden of cardiovascular and metabolic disorders among middle-aged and older population, driven by rapid urbanization, unique dietary habits (high refined carbohydrate intake, and low fiber) and genetic predispositions (24). The CHARLS is the first nationally representative survey focusing on this demographic that enables comprehensive research into the health of middle-aged and older Chinese adults. This study aims to characterize the distributions and temporal patterns, and associated factors of these six novel obesity indices in large Chinese cohort. Associated factors include demographic variables, socioeconomic factors, lifestyle behaviors, and medical conditions, providing a holistic perspective on obesity-related dynamics. The efforts will help build a foundation for the integration of these indices into future clinical screening, population health monitoring, and chronic disease risk stratification strategies tailored to Chinese populations.

## Methods

### Survey designs and populations

We utilized data from the CHARLS for 2011–2015. The initial survey, conducted between 2011 and 2012, included around 17,000 participants, with follow-up interviews in 2013, 2015, and 2018. CHARLS represents the first comprehensive, nationally representative study targeting residents aged 45 and older from 28 of the 31 provinces in mainland China (25). Using a multistage probability sampling method and face-to-face interviews, the survey achieved a response rate exceeding 80%. CHARLS offers the most current longitudinal datasets on health and functionality among middle-aged and older adults in China.

For our analysis, we used data from the first survey wave in 2011, focusing on a sample of 5,078 participants after excluding those missing certain covariates or obesity index components. Participants with acute or chronic diseases were not excluded. The cross-sectional analysis includes participants from the 2011 interviews, while the longitudinal analysis is based on 2,777 individuals who completed interviews in both 2011 and 2015. This study was approved by the ethics committee (IRB00001052-11015), and all participants provided written informed consent for participation in CHARLS.

### Measurement of novel obesity indexes

In this study, interviewers gathered anthropometric and other physical data, including waist circumference (WC), height, weight, triglycerides (TG), cholesterol, and high-density lipoprotein (HDL). WC was measured by trained interviewers using a soft tape placed horizontally over clothing at the level of the navel, with participants standing and holding their breath at the end of a normal exhalation. Morning venous blood samples were obtained after at least 8 h of overnight fasting, in accordance with CHARLS protocols. Biochemical analyses, including triglycerides and HDL-C, were performed using standardized enzymatic assays at certified local health centers under centralized quality control procedures. Based on available CHARLS data, six innovative obesity indices were calculated: VAI, CVAI, LAP, ABIS, WHtR, and WWI (15, 20, 26, 27).

VAI is a sex-specific composite index incorporating WC, BMI, TG, and HDL-C, used to estimate visceral fat function (Equations 1 and 2).

For men:


(1) $$\;\;\;{\mathrm{VAI}}_{\mathrm{men}}=(\frac{\mathrm{WC}}{39.68+1.88\times \mathrm{BMI}})\times (\frac{\mathrm{TG}}{1.03})\times (\frac{1.31}{\mathrm{HDL}-C})$$

For women:


(2) $$\;\;\;{\mathrm{VAI}}_{\text{women}}=(\frac{\mathrm{WC}}{36.58+1.89\times \mathrm{BMI}})\times (\frac{\mathrm{TG}}{0.81})\times (\frac{1.52}{\mathrm{HDL}-C})\;$$

CVAI is an index tailored to Chinese populations, designed to reflect visceral fat and metabolic syndrome risk. It includes age, WC, BMI, TG, and HDL-C. CVAI was calculated using Equations 3 and 4. For men:


(3) $$\begin{array}{l} {\text{CVAI}}_{\mathrm{men}}=-267.93+0.68\times \mathrm{age}+0.03\times \mathrm{BMI}\\ +\;4.0\times \mathrm{WC}(\mathrm{cm})+\;22\times \log10\mathrm{TG}-16.32\times \mathrm{HDL}-C \end{array}$$

For women:


(4) $$\begin{array}{l} {\text{CVAI}}_{\text{women}}=-187.32+1.71\times \mathrm{age}+4.23\times \mathrm{BMI}+\\ 1.12\times \mathrm{WC}(\mathrm{cm})+39.76\times \log10\mathrm{TG}-11.66\times \mathrm{HDL}-C \end{array}$$

LAP reflects the accumulation of triglyceride-rich fat in the abdominal area. LAP was calculated using Equations 5 and 6.


(5) $${\mathrm{LAP}}_{\mathrm{men}}=(\mathrm{WC}-65)\times \mathrm{TG}$$

(6) $$\;\;{\mathrm{LAP}}_{\text{women}}=(\mathrm{WC}-58)\times \mathrm{TG}$$

The ABSI adjusts waist size for height and BMI. It provides mortality risk estimation independent of total weight. ABSI was calculated using Equation 7.


(7) $$\;\;\text{ABSI}=\frac{\mathrm{WC}\;(m)}{{\mathrm{BMI}}^{2/3}\text{Height}\;{\left(m\right)}^{1/2}}\times 1000$$

WHtR is a simple screening tool for central obesity. A WHtR > 0.5 typically indicates increased health risk. WHtR was calculated using Equation 8.


(8) $$\text{WHtR}=\frac{\mathrm{WC}\;}{\text{Height}\;}$$

The WWI isolates abdominal fat relative to body mass. Higher WWI implies disproportionate fat distribution. WWI was calculated using Equation 9.


(9) $$\mathrm{WWI}=\frac{\mathrm{WC}\;(\mathrm{cm})}{\sqrt{\text{Weight}\;(\mathrm{kg})}}$$

### Independent variables

Age was divided into three categories 45–54 (reference), 55–64, and ≥65 years. Sex was classified as female (reference) and male. Marital status was grouped into living alone (reference) and living with a partner. Educational attainment was classified into five levels: illiterate (reference), less than elementary school, middle school or vocational school, and high school or above. Living status was grouped into rural (reference) and urban. Smoking status was classified as non-smoker (reference) or smoker. Drinking habits were grouped into non-drinker (reference) drinks less than once per month, and drinks more than once per month. Chronic disease status including diabetes, hypertension, and CKD, each coded as no (reference) and yes. These categorical variables were dummy-coded for regression analysis.

Hypertension was defined as (1) an average systolic blood pressure (SBP) of 140 mmHg or more, (2) an average diastolic blood pressure (DBP) of 90 mmHg or more, or (3) current use of anti-hypertensive medication (28). Diabetes criteria included (1) fasting plasma glucose of 126 mg/dL (7.0 mmol/L) or higher, (2) HbA1c level of 6.5% or higher, or (3) self-reported doctor-diagnosed diabetes or anti-diabetic medication use (29). CKD was identified by (1) an eGFR below 60 mL/min/1.73m^2^ (calculated using CKD-EPI equations) or (2) self-reported CKD (30).

### Statistical analysis

Analyses were conducted using SAS version 9.4 (SAS Institute, Cary, NC, US). Baseline characteristics stratified by age groups were systematically compared through appropriate parametric and nonparametric tests implemented in SAS modules: continuous variables were assessed with either one-way ANOVA (executed via PROC ANOVA) or Kruskal-Wallis test (using PROC NPAR1WAY), while categorical variables were examined with Pearson’s χ^2^ tests (implemented through PROC FREQ with CHISQ option), with distribution normality verified by Shapiro-Wilk tests. Linear regression models assessed the relationship between obesity indices and various covariates at baseline (CHARLS 2011) in the cross-sectional analysis.

To evaluate the association between changes in adiposity over time (CHARLS 2011 & 2015), generalized linear models with generalized estimating equations (GEE) were used, incorporating an unstructured correlation structure to account for intra-individual correlation from repeated measures. Parameter estimates were presented with 95% confidence intervals, and a two-sided *p*-value < 0.05 was considered statistically significant. The critical SAS code segments for index computation and regression modeling are available in the Supplementary material.

## Results

### Distribution of novel obesity indices based on CHARLS 2011

**Figure 1:**
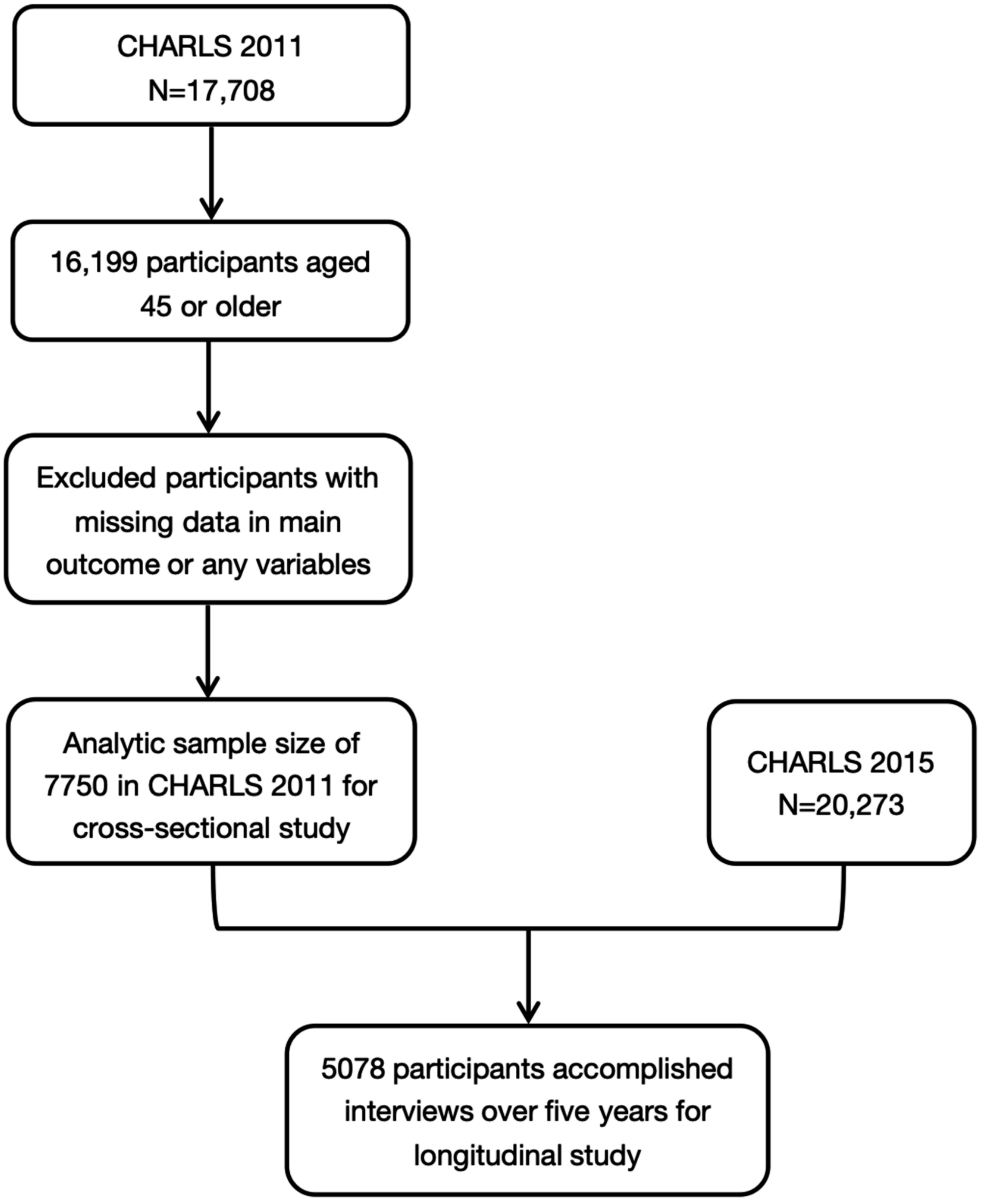
Sample screening of the present study. CHARLS, China Health and Retirement Longitudinal Study.

Based on the CHARLS, 17,708 Chinese adults over 45 were included at baseline in 2011, and 5,078 participants who completed all follow-up interviews from 2011 to 2015 were used for longitudinal analyses (Flowchart in Figure 1). Supplementary Table 1 shows the distributions of the main characteristics of the participants in the 2011 CHARLS survey. The mean VAI of the middle-aged and older population was 2.405 ± 4.21 (95% CI: 2.343–2.467), with a median of 1.48 (interquartile range [IQR]: 0.88–2.65) and a 5th -95th percentiles of 0.46 to 6.45. The ABSI had a mean of 0.083 ± 0.012 (95% CI: 0.0827–0.0831), a median of 0.082 (IQR: 0.075–0.090), and a narrower percentile range of 0.066 to 0.106. For WHtR, the mean was 0.542 ± 0.080 (95% CI: 0.540–0.544) and a median of 0.534 (IQR: 0.489–0.588), with percentiles spanning from 0.430 to 0.678. CVAI exhibited a higher degree of dispersion, with a mean of 98.025 ± 49.032 (95% CI: 97.009–99.041), median of 95.94 (IQR: 63.93–129.97), and percentile values ranging from 23.03 to 178.78. In contrast, WWI presented a relatively symmetric distribution, with a mean of 11.204 ± 0.940 (95% CI: 11.190–11.218), median of 11.13 (IQR: 10.58–11.78), and a range between 9.97 and 13.09. Lastly, LAP demonstrated the widest variability, with a mean of 38.83 ± 41.988 (95% CI: 37.96–39.70), a median of 26.01 (IQR: 14.44–46.46), and a 5th–95th percentile span from 4.80 to 123.18. Overall, these indices demonstrated a consistent upward trend with increasing age. Notably, across all age groups, females exhibited significantly higher values than males for each index. These sex differences were particularly pronounced for CVAI, LAP, and VAI.

### Associated factors with obesity indices

To identify potentially determinates, we first conducted univariate linear regression analyses, which revealed that age, gender, marital status, education, living area, lifestyle habits (smoking and drinking), and chronic illness (diabetes, hypertension, and CKD) were significantly associated with these obesity indices (Supplementary Table 2). To further delineate these relationships, multivariate line*a*r regression analyses were conducted (Supplementary Table 3). Gender and diabetes still significantly impacted all six novel obesity indexes after being adjusted by other confounders (all *p* values <0.05). In contrast, the associations of other factors—such as marital status, education, living status, smoking, drinking, and CKD—were attenuated, with fewer indices remaining significantly affected, indicating that their effects may have been partially confounded. Hypertension and age continued to exhibit prominent and relatively consistent effects across multiple obesity indices. BMI showed varying impacts: it was positively associated with WHtR (*β* = 0.00403, *p* < 0.01), CVAI (*β* = 1.693283, *p* < 0.01), and LAP (*β* = 0.29421, *p* < 0.01), while it negatively impacted ABSI (*β* = −0.00018, *p* < 0.01).

### Longitudinal trends in novel obesity indices from CHARLS 2011 to 2015

**Figure 2:**
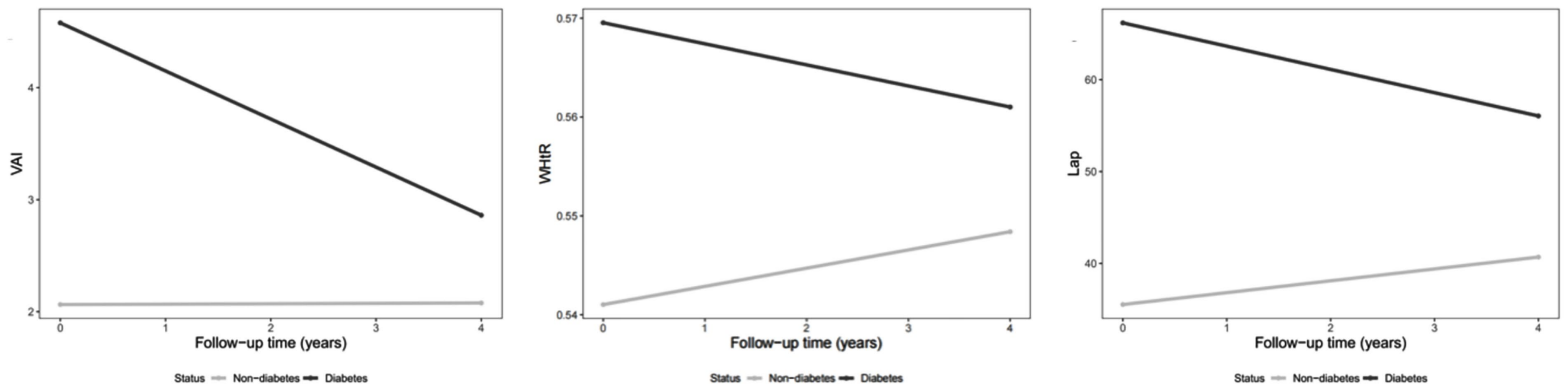
Changes in obesity indexes over time (2011–2015) among diabetes patients compared to non-diabetes patients.

Supplementary Table 4 summarized the GEE results of baseline factors were associated with longitudinal changes in six novel obesity indices over a four-year period. Consistent with cross-sectional analyses, several baseline characteristics, including gender, diabetes, and hypertension, remained significantly associated with the longitudinal trends in all six novel obesity indices from 2011 to 2015. Specially, male was negatively associated with these indices (VAI: *β* = −1.09; ABSI: *β* = −0.002; WHtR: *β* = −0.038; CVAI: *β* = −9.860; WWI: *β* = −0.686; and LAP: *β* = −17.695). In contrast, both diabetes and hypertension at baseline were positively associated with increases in all obesity indices over time among middle-aged and older adults. More importantly, baseline BMI exhibited divergent longitudinal associations with different adiposity indices: higher BMI at baseline was significantly associated with a decrease in ABSI over time (*β* = −0.00015, *p* < 0.01), whereas it was positively associated with an increase in WHtR (*β* = 0.00403, *p* < 0.01) over the 4-year follow-up period. We observed that WHtR, CVAI, and LAP increased over time, whereas ABSI showed a potential decrease as time progressed. Notably, VAI, WHtR, and LAP remained significant even without adjusting for confounders (as shown in Figure 2). As for CKD and hypertension, this kind of trend did not exist. Notably, VAI (Figure 2A), WHtR (Figure 2B), and LAP (Figure 2C) decreased over time among diabetes patients compared to non-diabetes patients, as shown is Figures 2A,B,C. However, similar trends were not observed among patients with CKD or hypertension.

### Subgroup analysis of aging population with diabetes

A subgroup analysis was conducted among participants with diabetes, given its high prevalence and strong association with obesity in Chinese aging population. Compared with non-diabetic individuals, participants with diabetes exhibited significantly different longitudinal trends in obesity indices. Over the follow-up period from 2011 to 2015, diabetic individuals showed decreases in VAI (*β* = −1.693, 95% CI: −2.421 to −0.965), WHtR (*β* = −0.009, 95% CI: −0.015 to −0.004), CVAI (*β* = −5.408, 95% CI: −10.509 to −0.308), and LAP (*β* = −14.989, 95% CI: −20.879 to −9.100). These findings suggest that the temporal changes in visceral adiposity differed substantially among diabetic individuals, indicating distinct obesity trajectories and possibly differential metabolic responses over time.

## Discussion

This population-based study is the first to systematically examine the national ranges and longitudinal changes of six novel obesity indices—VAI, WHtR, WWI, ABSI, CVAI, and LAP— among middle-aged and older Chinese adults. We also investigated how demographic, lifestyle, and health-related factors influence these indices over time. Rather than evaluating disease outcomes directly, our study provides foundational epidemiological evidence that characterizes how these indices behave in a real-world aging population. These findings lay the groundwork for future longitudinal and clinical studies to assess their potential predictive value and inform their application in public health and clinical risk assessment.

Obesity can be broadly categorized into general obesity and central obesity. While BMI is widely used to assess general obesity (31), has significant limitations in capturing fat distribution, particularly in aging populations where fat tends to accumulate in the abdominal region despite stable BMI (4). Furthermore, BMI does not differentiate between fat-free mass and fat mass, often leading to misclassification, especially in individuals with excess visceral fat or chronic conditions like diabetes (32). This underscored the importance of novel obesity indexes for accurately assessing central obesity and predicting metabolic and cardiovascular risks.

### WHtR, ABSI and WWI

WHtR, ABSI, and WWI are key metrics for assessing central obesity and body shape. In this study, the WHtR range and WWI range align with international findings, including Iranian research, which identified optimal cut-offs for Metabolic Associated Fatty Liver Disease (e.g., WHtR: 0.56 for males, 0.63 for females; WWI: 10.63 for males, 11.71 for females), suggesting good cross-population applicability (33). The higher ABSI range in this study compared to previous research on younger Chinese individuals may reflect age differences, as our population (aged 45 and above) likely has a higher prevalence of obesity (34).

Significant factors influencing the three obesity indexes included age, gender, marital status, urban living, health conditions, and other lifestyles. Older individuals and females consistently showed higher values in these indexes, reflecting increased abdominal fat accumulation and changes in body composition with aging. The observed differences in females are likely due to due to hormonal and metabolic factors, particularly post-menopause (35). WHtR, in particular, was influenced by marital status, place of residence, diabetes, and smoking, suggesting its greater susceptibility to external factors compared to ABSI and WWI, although the effects varied. Frequent alcohol intake was positively associated with ABSI, reflecting the potential role of alcohol in promoting abdominal fat deposition and altering body shape (36). BMI was negatively associated with ABSI while positively associated with WHtR due to their reliance on WC measurements, which are inherently related to BMI. While WHtR captured abdominal obesity, ABSI adjusted for height and weight, focusing more on body shape and central adiposity (37). However, WWI, which accounts for WC relative to weight, also showed no significant association with BMI, highlighting the limitations of BMI in capturing central obesity and metabolic dysfunction (38).

Our study observed that WHtR increased over time, whereas ABSI showed a potential decrease. This trend indicated that central obesity and associated risks may intensify with aging. As these indexes were closely linked to metabolic and cardiovascular risks, their upward trend highlights the need for targeted interventions to address central obesity and its related health risks in older adults. The observed decrease in ABSI may result from its unique calculation, which adjusts waist circumference for height and BMI. This decline could be influenced by age-related reductions in height or population-level BMI trends, which disproportionately affect ABSI.

These findings suggest that WHtR and WWI may serve as practical tools in clinical settings for monitoring central obesity in older adults. Given their simplicity and independence from body weight, these indices can be readily applied in routine physical examinations. Physicians may use WHtR or WWI cut-off points to flag patients who require lifestyle interventions or further cardiometabolic screening, particularly in cases where BMI appears normal but central adiposity is suspected.

### Lap, VAI and CVAI

The observed ranges for CVAI and LAP aligned closely with those reported in another Chinese study on hyperuricemia patients aged 40 and above, while the VAI range showed notable differences (39). CVAI has demonstrated greater utility in assessing abdominal obesity and cardiometabolic risk, particularly in T2DM patients in China, while VAI may require refinement for broader applicability (17, 40). Importantly, while LAP and CVAI were originally developed to reflect visceral fat accumulation and insulin resistance, emerging evidence suggests they may also partly capture hepatic steatosis. Hepatic fat accumulation, independent of visceral obesity, can contribute to systemic insulin resistance via hepatokine-mediated mechanisms (41). Therefore, these indices may reflect overlapping but distinct aspects of ectopic fat, and caution should be exercised when interpreting their metabolic implications.

The three obesity indexes were associated with a range of demographic, medical, and lifestyle factors, including age, sex, urban living, and health conditions. CVAI increased with age, reflecting greater abdominal fat accumulation and altered body composition, while LAP decreased, potentially due to age-related changes in lipid metabolism or lower triglyceride levels. Notable sex differences were observed, with females showing higher values of VAI, CVAI, and LAP, consistent with findings for WHtR, WWI, and ABSI. Living with a partner and residing in urban areas were both associated with higher CVAI and LAP values, suggesting that social and environmental factors, such as shared dietary patterns, reduced physical activity, sedentary behaviors, and increased access to calorie-dense foods, may contribute to central obesity (42, 43).

Diabetes and hypertension were significantly associated with increased obesity indexes, particularly LAP and CVAI, which effectively capture visceral fat accumulation. Visceral fat contributes to chronic low-grade inflammation and metabolic dysregulation, which are key pathways driving both IR and elevated blood pressure (44, 45). In contrast, CKD showed no significant associations with obesity indexes, differing from previous studies that linked CKD to visceral fat and lipid metabolism (46). This discrepancy may stem from differences in CKD severity, population characteristics, or the influence of non-obesity-related factors like oxidative stress and inflammation. In addition, BMI showed no significant associations with VAI, CVAI, and LAP, as these indexes were specifically designed to measure visceral fat and lipid metabolism using metabolic parameters like triglycerides and HDL cholesterol (40). These findings highlight the limitations of BMI in reflecting central obesity and emphasize the need for advanced metrics to better evaluate obesity-related risks in diverse populations.

Interestingly, participants with diabetes showed decreases in VAI, CVAI, and LAP over time, likely reflecting advanced metabolic dynamics associated with disease progression. In later stages, poorly controlled diabetes can lead to unintentional weight loss and muscle wasting due to impaired glucose utilization and increased reliance on fat and protein for energy. These catabolic effects could contribute to reductions in visceral fat and triglyceride levels, which directly impact these obesity indexes (6). Moreover, diabetes-related complications, such as CKD or CVD, may exacerbate metabolic consumption, further reducing lipid reserves and central adiposity. In addition, the observed decreases may also be partially attributed to effective disease management strategies, including lipid-lowering therapy, dietary modifications, physical activity, and improved glycemic control. These interventions can reduce central adiposity and improve metabolic parameters, thereby lowering obesity indices over time. While these decreases may suggest metabolic improvements, they could also signify underlying metabolic deterioration, particularly in patients experiencing significant weight or fat loss because of long-term disease progression. This duality underscored the importance of interpreting obesity index trends in the context of both disease management and progression.

Given the strong associations between LAP and CVAI with diabetes and hypertension observed in our study, these indices may be integrated into risk stratification algorithms for older adults. For example, elevated LAP values may prompt physicians to initiate early screening for insulin resistance or metabolic syndrome. Similarly, high CVAI levels in routine assessments could support timely interventions such as dietary modifications, triglyceride-lowering therapies, or personalized exercise programs targeting visceral fat. These indices may thus serve as actionable indicators to guide individualized care and chronic disease prevention.

This study is the first to examine the distribution and variation of novel obesity indices and their associated factors in middle-aged and older Chinese adults, addressing the BMI-based obesity paradox and providing a more accurate assessment of body adiposity. Using data from the nationally representative CHARLS survey, we ensured the generalizability of our findings and adjusted for multiple confounders. However, the reliance on self-reported data, such as smoking and drinking habits, may introduce reporting bias, a limitation inherent to large-scale studies like NHIS and NHANES.

## Conclusion

In conclusion, this study provided comprehensive insights into the factors influencing novel obesity indexes among middle-aged and older Chinese adults. The findings underscored the critical roles of demographic, lifestyle, and health-related factors in shaping obesity distribution and trends. The strong associations with diabetes, hypertension, and CKD underscore the need for integrated management strategies to address obesity-related risks. The observed time trends and interaction effects emphasized the dynamic nature of obesity, calling for sustained public health efforts to mitigate its growing burden.

## Data Availability

The datasets presented in this study are publicly available. This study represents a secondary analysis utilizing CHARLS public data, which are accessible globally at http://charls.pku.edu.cn/.
