# Supplementary material for: Ranges, temporal trend, and determinants of novel obesity indices in middle-aged and older Chinese population: insights from a nationally representative and longitudinal study
Source: Front Public Health. 2025 Aug 14;13:1571254. doi: 10.3389/fpubh.2025.1571254 (PMC12390798; doi:10.3389/fpubh.2025.1571254)
Supplement: Supplementary file 5 [file Table_5.DOCX]

/* CALCULATION OF NOVEL OBESITY INDICES (VAI, CVAI, ABSI, LAP, WWI)*/

**data** final;

set cohort_data; /* Based on China Health and Retirement Longitudinal Study (CHARLS) */

tg_mmol = tg * **0.01129**; /* TG: mg/dL → mmol/L */

hdl_mmol = hdl * **0.02586**; /* HDL: mg/dL → mmol/L */

height_m = ht / **100**; /* Height: cm → m */

if sex = **1** then do; /* Male */

vai = (wc / (**39.68** + **1.88***bmi)) * (tg_mmol/**1.03**) *(**1.31**/hdl_mmol);

cvai = -**267.93** + **0.68***age + **0.03***bmi + **4.00***wc + **22.0***log10(tg_mmol) - **16.32***hdl_mmol;

lap = (wc - **65**) * tg_mmol;

end;

else if sex = **2** then do; /* Female */

vai = (wc / (**36.58** + **1.89***bmi)) * (tg_mmol/**0.81**) *(**1.52**/hdl_mmol);

cvai = -**187.32** + **1.71***age + **4.23***bmi + **1.12***wc + **39.76***log10(tg_mmol) - **11.66***hdl_mmol;

lap = (wc - **58**) * tg_mmol;

end;

bmi = bw / (height_m****2**); /* kg/m² */

whtr = wc / ht; /* (cm/cm) */

absi = (wc/**100**) / (bmi**(**2**/**3**) * sqrt(height_m));

wwi = wc / sqrt(bw);

**run**;

**data** strata;

set final;

length age_group $**10**;

if age >=**45** & age<=**54** and sex=**1** then age_group = "45-54_M";

else if age >=**55** & age<=**64** and sex=**1** then age_group = "55-64_M";

else if age >=**65** and sex=**1** then age_group = "65+_M";

else if age >=**45** & age<=**54** and sex=**2** then age_group = "45-54_F";

else if age >=**55** & age<=**64** and sex=**2** then age_group = "55-64_F";

else if age >=**65** and sex=**2** then age_group = "65+_F";

**run**;

**proc** **univariate** data = strata normal;

class age_group;

var vai;

**run**;

**proc** **anova**;

class age_group;

model vai = age_group;

**run**;

**proc** **reg** data = final outest = parm tableout;

model vai = age sex1 edu1 edu2 edu3 edu4 marry1 smoke1 drink1 drink2 urbanrural1 ckd1 db hypertension bmi;

**run**;

**proc** **genmod** data=long_format;

class

id

sex (ref='2')

edu (ref='1')

marry (ref='2')

smoke (ref='2')

drink (ref='3')

urbanrural (ref='1')

db (ref='0')

hypertension(ref='0')

ckd (ref='0')

;

model vai = age sex edu marry smoke drink urbanrural ckd db hypertension bmi time time*ckd time*db time*hypertension / link=identity;

repeated subject=id / corr=unstr;

**run**;
